# The access route through the anatomical snuffbox in ultrasound-guided synovial biopsy of the wrist allows for a safe and effective collection of tissue samples in inflammatory arthritis

**DOI:** 10.1186/s13075-023-03101-y

**Published:** 2023-07-08

**Authors:** Ludovico De Stefano, Serena Bugatti, Veronica Piccin, Gioacchino D’Ambrosio, Terenzj Luvaro, Blerina Xoxi, Carlomaurizio Montecucco, Antonio Manzo

**Affiliations:** 1 Department of Internal Medicine and Therapeutics, Università Di Pavia, Pavia, Italy; 2 Division of Rheumatology, Fondazione IRCCS Policlinico San Matteo, Viale Golgi 19, 27100 Pavia, Italy; 3 Unit of Anatomic Pathology, Fondazione IRCCS Policlinico San Matteo, Pavia, Italy

**Keywords:** Synovial biopsy, Synovitis, Ultrasound, Wrist, Small joints, Arthritis

## Abstract

**Background:**

A proof-of-concept study to evaluate the feasibility and safety of minimally invasive ultrasound (US)-guided synovial biopsy of the radiocarpal (RC) joint using the anatomical snuffbox as an access route.

**Methods:**

Twenty consecutive patients with active chronic arthritis of the wrist underwent minimally invasive US-guided synovial biopsy of the RC joint using the anatomical snuffbox as the access route. Samples were retrieved from 3 predetermined biopsy target sites of the RC synovia (proximal, vault, and distal site), aiming for a minimum of 12 samples. The procedure’s feasibility was evaluated based on the number and histological quality of retrieved tissue fragments tested on pre-defined histometric parameters. The safety and tolerability of the procedure were assessed through 1-week and 1-month follow-up clinical evaluations.

**Results:**

A median number of 17 fragments (≥ 1 mm diameter size at macroscopic evaluation) per procedure was processed for histopathology (range 9–24) and dedicated to the study. At the histopathologic evaluation, a gradable tissue (visible lining layer and ≥ 4 fragments with IST) was recognized in 19/20 biopsies (95%), and all pre-defined histometric parameters were judged applicable and successfully measured in 19/19 gradable biopsies. All three biopsy target sites showed sampling accessibility. The entire procedure was generally well tolerated. At the 1-month follow-up, no patients showed infectious complications.

**Conclusions:**

The access route through the anatomical snuff box in US-guided synovial biopsies of the RC joint allows for a safe and targeted collection of adequate tissue samples. This modification of the traditional access route may allow easier, repeatable, and safer sampling of anatomically distinct areas of the wrist in the course of arthritis.

## Background

Clinical research on synovial tissue analysis in patients with chronic arthritis has increased in recent years. Interest is mainly focused on studying the early stages of the disease, not so much as a diagnostic tool [1], but on understanding pathogenic mechanisms and obtaining additional markers helpful for guiding therapeutic strategies [2–7].

In order to promote the widespread inclusion of synovial tissue analysis into clinical practice and research, samples need to be acquired mini-invasively and from representative joints. The development and validation of ultrasound (US)-guided techniques, using either a semi-automatic core needle or a portal and forceps approach, has offered an alternative tool to arthroscopy with excellent outcomes in terms of safety and accessible joints [8–10]. Still, sampling from the small joints of the hands, which are the most frequent and early target of the inflammatory process in many idiopathic arthritis including rheumatoid arthritis (RA), although feasible [11, 12], remains challenging due to potentially inadequate tissue yield. In this context, the radiocarpal (RC) joint offers the most suitable alternative, being larger and containing a greater amount of synovium [8, 12]. Usually, access to the RC joint, whether to perform intra-articular injections [13], arthroscopies [14], or synovial biopsies [15], is performed between the extensor tendons compartments III and IV, IV and V, and V and VI on the dorsal side. Nonetheless, in our experience, the anatomical characteristics of the RC joint and the course of the various periarticular structures, particularly the tendons, may condition the path and the inclination of the surgical instrument within the joint cavity, with the possibility that part of the synovial surface remains unreachable.

The anatomical snuff box is a triangular deepening that forms when the thumb is extended and abducted on the radial side of the wrist at the level of the carpal bones. The deepening is bounded medially by the tendon of the extensor pollicis longus muscle, laterally by the tendons of the extensor pollicis brevis and the abductor pollicis longus, distally by the base of the I metacarpal bone and proximally by the styloid process of the radius; the scaphoid and trapezius bones and the medial side of the RC joint define the floor [16]. In this area, the needle can be introduced through the joint capsule. It can be directed in the radial-ulnar direction, parallel to both the cartilaginous surface lining the carpal bones (scaphoid, lunate, and part of the pyramid bone) and the synovial lining of the upper side of the joint capsule extended between the dorsal border of the radius and the contour of the carpal condyle, until it reaches the triangular ligament of the carpus.

Despite this potentially favorable substrate, no data are currently available in the literature on the specific output of synovial biopsy of the wrist based on the above-described anatomical approach. In light of these considerations, we designed a proof-of-concept study to evaluate the feasibility and safety of RC synovial biopsy using the anatomical snuffbox as the access route.

## Methods

### Patient inclusion

The study, data collection, and reporting were performed according to the EULAR points to consider for minimal reporting requirements in synovial tissue research in rheumatology [17]. Twenty consecutive patients undergoing US-guided needle synovial biopsy of the wrist at the Division of Rheumatology of the IRCCS Policlinico San Matteo Foundation of Pavia between February 2021 and December 2022 were included. Indications for synovial biopsy were clinically unspecified synovitis or persistent unexplained arthritis in patients with an established diagnosis. The study was conducted according to the Declaration of Helsinki. All patients signed informed consent before inclusion, and the local Ethics Committee approved the study protocol.

### US evaluation and US-guided synovial biopsy technique

US examination (in gray scale, GS, and power Doppler, PD) and US-guided synovial biopsy were performed using an Esaote MyLab™Seven machine with a linear probe (6/18 MHz). Synovial thickening and degree of PD signal of the wrist were scored semi-quantitatively (0–3) [18] immediately before the biopsy procedure. In each patient, the RC joint with the highest US synovitis score was selected for biopsy.

**Fig. 1:**
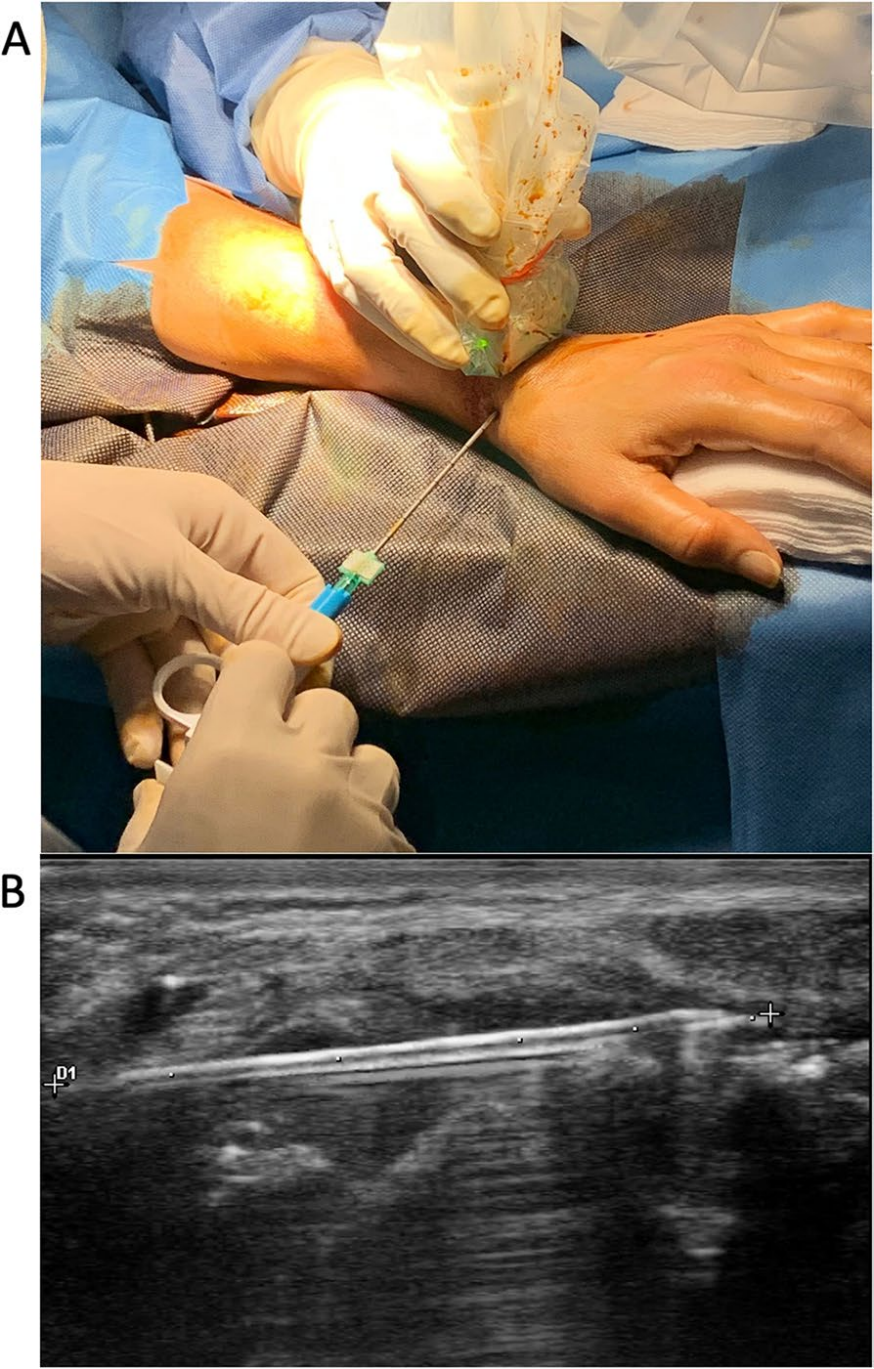
Ultrasound (US)-guided synovial biopsy of the radiocarpal (RC) joint using the anatomical snuffbox as the access route. **A** A representative image of a patient undergoing US-guided RC synovial biopsy using a Quick-Core needle (14Gx100 mm). Access is located, with the hand pronated, wrist in slight extension, and thumb abducted, at the bottom of the anatomical snuffbox, immediately below the extensor pollicis longus tendon, superior to the lateral RC ligament, and approximately 5 mm proximal to the trapezius. **B** US guidance allowed the needle to penetrate approximately 3 cm into the RC joint until it reached the triangular ligament of the carpus. The needle can be visualized in its entire length as a small hyperechoic line within the synovial proliferation above the carpal bones

Two trained operators (LDS [procedure] and SB [US assistance], with > 5 years of experience in US-guided biopsy) performed all biopsy procedures. All biopsy procedures were carried out on an outpatient basis in a sterile environment. Access was localized, with the hand pronated, wrist in slight extension, and thumb abducted, at the bottom of the anatomical snuffbox, immediately below the extensor pollicis longus tendon, superior to the lateral RC ligament and approximately 5 mm proximal to the trapezius (Fig. 1A). The skin and subcutaneous tissue of the anatomical snuffbox over the joint capsule were infiltrated with 5 mL of local anesthetic (1% lidocaine) using a 25-gauge needle. A further 5 mL of local anesthetic (1% lidocaine) was instilled into the synovial space. After 2 min, a Quick-Core biopsy needle (14Gx100 mm) was inserted into the synovial space under direct US vision. The needle could be dynamically visualized in its entire length as a small hyperechoic line within synovial proliferation above the carpal bones (Fig. 1B).

US guidance allowed the needle to penetrate approximately 3 cm into the RC joint until it reached the triangular ligament of the carpus (Fig. 1B). The needle, parallel to the carpal cartilaginous floor, remained about 3–4 mm away from it throughout its journey. In contrast, it made direct contact with the synovial tissue lining the dorsal side of the joint capsule. The needle could then be angled dorsally, about 20°, and laterally, carpal and radial, at an angle of about 30°. In this way, with the tip of the needle, it was possible to reach any point of the synovium lining the vault of the joint capsule extended between the dorsal edge of the articular surface of the radius and the dorsal edge of the carpal condyle, with the only exception of the most proximal 5 mm of the radio-scaphoid joint.

**Fig. 2:**
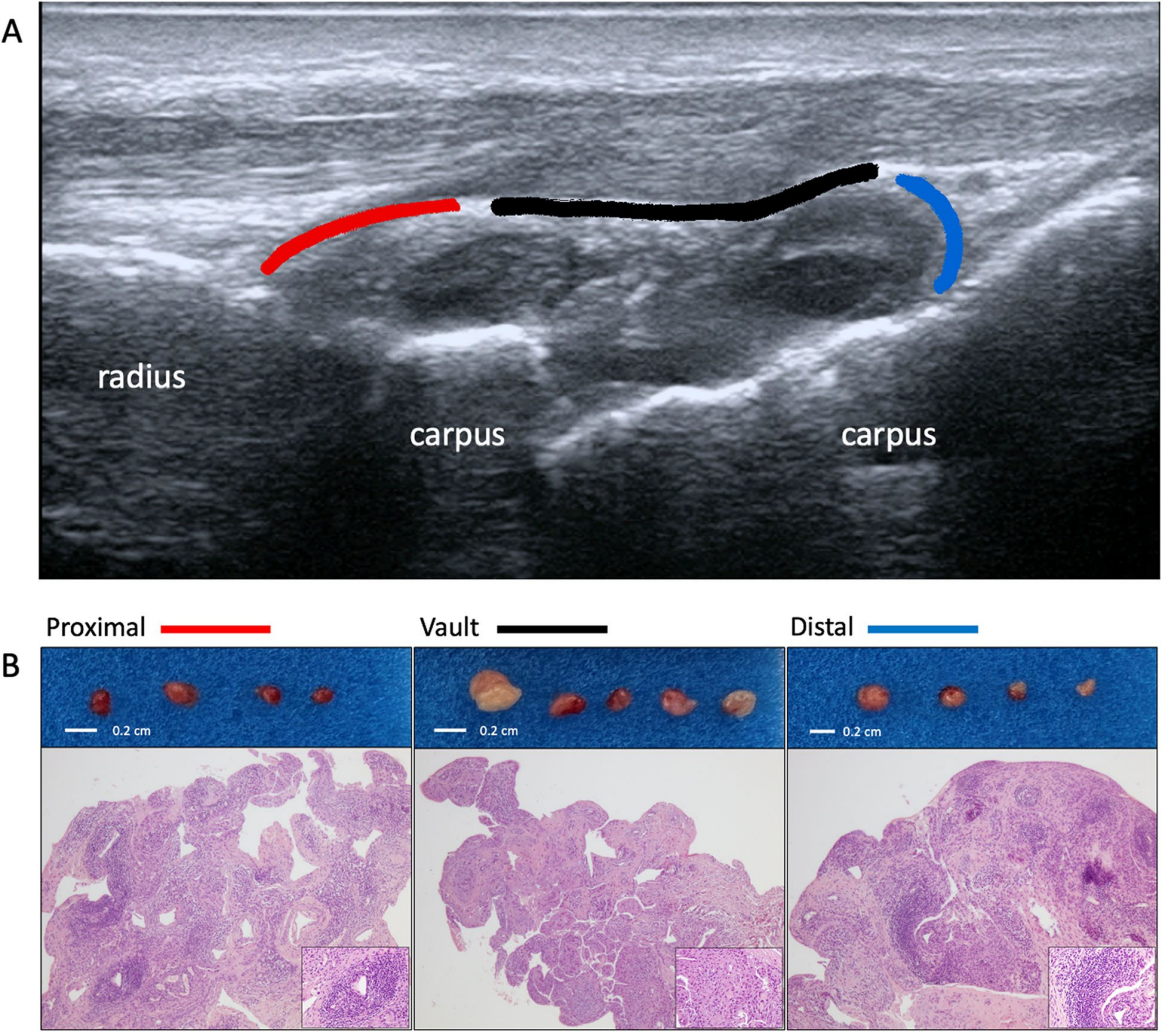
Target sites for radiocarpal (RC) synovial biopsy and retrieved tissue fragments. **A** Representative ultrasound longitudinal scan of the radiocarpal joint. The perimeter of the radiocarpal synovitis is marked in different colors to indicate the 3 target sites used in the collection of biopsy specimens. In red is the proximal site, in black is the vault site, and in blue is the distal site. **B** Representative images of retrieved synovial tissue fragments for each target site. Macroscopic and histological images stained with hematoxylin and eosin (at × 10 magnification and detail of the lymphocyte infiltrate at × 20 magnification) of representative synovial tissues for each target site

Predetermined biopsy target sites were (i) the articular surface of the radius (proximal site), (ii) the articular surfaces of the carpal bones (distal site), and (iii) the vault of the joint capsule, especially where the synovium appeared thicker (vault site) (Fig. 2A). The maximum tolerated number of biopsies per joint was attempted, aiming for a minimum of 12 samples (≥ 1 mm diameter size at macroscopic evaluation) to be retrieved among the three target sites and through a target procedural time of 30 min [12].

### Synovial tissue processing

Biopsy specimens were kept moist in saline for a maximum of 30 min after completion of the procedure and numbered macroscopically (retrieved tissue fragments). A variable number of fragments (depending on the total number and concomitant downstream procedures) was randomly selected for histopathology and fixed in 4% formalin for up to 24 h. After paraffin embedding, 5-μm-thick serial sections were cut using a standard microtome, mounted onto glass slides, and stained with hematoxylin and eosin (H&E) according to the standard protocols.

**Table 1:** Ultrasound and histological data

**Biopsy code, *n*°**	**GS score**	**PD score**	**Fragments H&E, *n* total/IST (TQR)**	**Biopsy gradable**^**a**^	**Krenn score (synovitis grade)**	**Vascularity score**	**Cell aggregation G2/G3 (LAI)**
1	3	2	12/9 (75%)	+	6.5 (high)	1	+ / + (0.89)
2	2	2	16/0 (0%)	−	*ne (ne)*	*ne*	*ne (ne)*
3	2	2	21/14 (67%)	+	3 (low)	0.5	+ / + (0.14)
4	2	3	14/14 (100%)	+	6 (high)	1.5	+ / + (0.86)
5	2	1	23/22 (96%)	+	5.5 (high)	0	+ / − (0.14)
6	1	2	16/5 (31%)	+	4 (low)	0.5	− / − (0.00)
7	3	3	13/12 (92%)	+	4.5 (low)	1	+ / − (0.33)
8	2	3	19/9 (47%)	+	4.5 (low)	0.5	+ / − (0.22)
9	3	2	12/12 (100%)	+	7.5 (high)	2	+ / + (0.50)
10	3	3	20/9 (45%)	+	4.5 (low)	1	− / − (0.00)
11	1	3	9/7 (78%)	+	5 (high)	0.5	+ / − (0.37)
12	2	2	10/8 (80%)	+	5 (high)	1.5	+ / + (0.62)
13	2	1	17/6 (35%)	+	3.5 (low)	0.5	− / − (0.00)
14	2	2	24/18 (75%)	+	5.5 (high)	2	+ / − (0.18)
15	3	2	19/10 (53%)	+	4 (low)	0	− / − (0.00)
16	2	3	20/20 (100%)	+	6 (high)	1.5	+ / + (0.87)
17	2	3	18/16 (89%)	+	4.5 (low)	1	+ / + (0.59)
18	3	3	18/18 (100%)	+	6.5 (high)	2.5	+ / + (0.52)
19	3	2	16/12 (75%)	+	5.5 (high)	1.5	+ / − (0.23)
20	2	1	18/13 (72%)	+	4.5 (low)	0.5	+ / − (0.16)

*GS* gray scale of the biopsied wrist, *PD* power Doppler of the biopsied wrist, *Fragments H&E* fragments selected for histopathology, *IST* intact synovial tissue, *TQR* tissue quality ratio, *G2/G3* grade 2/grade 3 cell aggregates, *LAI* lymphocyte aggregation index, *ne* not evaluable

^a^Characterized by a visible lining layer and ≥ 4 fragments displaying IST

### Study outcomes

The following co-primary outcomes were considered.

#### Feasibility

Procedural feasibility was defined as the retrieval of histologically assessable tissue. Each biopsy was evaluated on H&E slides at a single cutting level to identify the fragments characterized by intact synovial tissue (IST, defined microscopically by the presence of characteristic vessels and stroma in the absence of artefactual changes) [5]. Biopsies were defined as gradable if containing a visible lining layer and a number of fragments displaying IST ≥ 4 [8]. For descriptive purposes, a tissue quality ratio (TQR) was then assigned to each biopsy according to the proportion of the fragments displaying IST/total fragments in the section.

The applicability of different histometric analyses to gradable biopsies was tested directly through the quantification of Krenn’s score [19], sub-lining vascularity (semi-quantitatively, 0–3) [20], and cell aggregation (measured dichotomously in terms of the presence/absence of grade (G) 2/G3 lymphocyte aggregates [21] and as an index [lymphocyte aggregation index, LAI] expressing the ratio between the total number of G2 and -G3 aggregates/total number of fragments displaying IST).

All tissue assessments were performed blindly by two independent readers with > 5 years of experience in synovial tissue analysis (TL and AM). Discrepancies were solved by mutual agreement or by calculating the mean score (Krenn’s and vascularity semi-quantitative scores).

#### Safety and tolerability

After the synovial biopsy, each patient was followed up at 7 and 30 days through direct clinical evaluation to assess the number and type of adverse events and/or local pain worsening. Joint pain was measured pre-biopsy, immediately post-biopsy, after 1 week and 1 month using a visual analog scale (VAS) ranging from 0 to 100 mm. Any worsening of ≥ 20 mm compared to the pre-biopsy assessment could be hypothesized to be related with the biopsy procedure. Short-term safety and tolerability were defined as the absence of adverse events and pain worsening over the selected time points compared to pre-biopsy.

## Results

Ten patients had early arthritis (symptoms duration £ 12 months), 8 had established RA (symptoms lasting > 5 years), and 2 had arthritis in the course of cancer immunotherapy with checkpoint inhibitors. Thirteen out of 20 patients were females, the average age was 45 years (SD ± 7.9), and there were no obese patients (mean BMI value 24, SD ± 4.2). None had received glucocorticoid injections of the biopsied wrist in the previous 4 weeks. All patients had clinical signs of joint inflammation in the biopsied wrist (swelling and tenderness) and at least grade 2 synovitis of the RC joint at US examination [18, 22]. The mean (range) 28-Joints Disease Activity Score (DAS28) of the study population was 3.99 (2.77–5.07).

A median number of 17 fragments (≥ 1 mm diameter size at macroscopic evaluation) per procedure was processed for histopathology (range 9–24) and dedicated to the study (Table 1). At the histopathologic evaluation, a gradable tissue (visible lining layer and ≥ 4 fragments with IST) was recognized in 19/20 biopsies (95%) displaying a median TQR of 75% (range 31–100) (Table 1). All three pre-defined histometric parameters were judged applicable and successfully measured by both study readers in 19/19 gradable biopsies (Table 1).In the first five recruited patients with early arthritis (selected pre-biopsy and pre-US examination), the anatomic origin of the retrieved fragment was tracked to explore the accessibility and tissue quality in the 3 targeted wrist sites.

All three sub-compartments (proximal, distal, and vault) showed sampling accessibility with the 14-G biopsy needle. The histological analyses demonstrated feasibility in retrieving gradable tissue and detecting pathologic changes in all targeted wrist areas (Fig. 2B and Table 2).

**Table 2:** Histological analysis of wrist sub-compartments

**Biopsy code, *n***	**Biopsy site**	**Krenn score (synovitis grade)**	**Vascularity score**	**Cell aggregation G2/G3 (LAI)**
1	D^a^	*ne (ne)*	*ne*	*ne (ne)*
	V	6.5 (high)	1	− / + (0.50)
	P	7 (high)	1.5	+ / − (1.50)
4	D	6 (high)	1.5	+ / + (1.50)
	V	4 (low)	1	− / − (0.00)
	P	6 (high)	2	+ / + (1.20)
5	D	5.5 (high)	1	+ / − (0.11)
	V	4 (low)	0.5	+ / − (0.15)
	P^b^	*ne (ne)*	*ne*	*ne (ne)*
8	D	5 (high)	1	+ / − (0.39)
	V	4 (low)	0	− / − (0.00)
	P	4.5 (low)	0.5	+ / − (0.38)
12	D	6.5 (high)	2.5	+ / + (0.89)
	V	4 (low)	0.5	+ / − (0.25)
	P	5.5 (high)	1.5	+ / + (0.73)

*D* articular surface of the carpal bones (distal site), *V* vault of the joint capsule (vault site), *P* articular surface of the radius (proximal site), *G2/G3* grade 2/grade 3 cell aggregates, *LAI* lymphocyte aggregation index, *ne* not evaluable

^a^Non-gradable (< 4 fragments with intact synovial tissue at the section level analyzed)

^b^Not performed due to patient discomfort

**Fig. 3:**
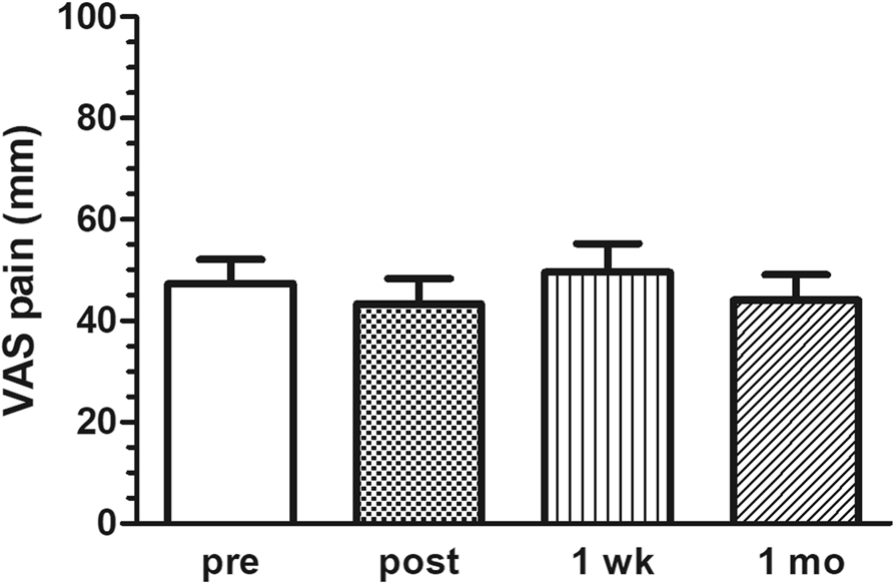
Joint pain as a measure of safety and tolerability of the biopsy procedure. Pain at the biopsy site was measured pre-biopsy (*pre*), immediately post-biopsy (*post*), after 1 week (*1 wk*), and 1 month (*1 mo*) using a visual analog scale (*VAS*) ranging from 0 to 100 mm. Any worsening of ≥ 20 mm compared to pre-biopsy assessment was interpreted as secondary to the procedure. As shown in the figure, pain scores showed non-significant changes with a mean (SD) ΔVAS of − 4 (7) immediately after biopsy, 2.3 (12) at the 1 week, and − 3.3 (3.9) at the 1 month review

The entire procedure was generally well tolerated. All patients reported mild discomfort only during skin, subcutis, and joint capsule anesthesia. Pain scores showed non-significant changes with a mean (SD) ΔVAS of − 4 (7) immediately after biopsy, 2.3 (12) at the 1 week, and − 3.3 (3.9) at the 1-month review (Fig. 3). Worsening joint pain (ΔVAS pain ≥ 20) occurred in 2/20 (10%) of the patients immediately post-biopsy and in none after 1 week and after 1 month. In all cases, the pain was managed with analgesics and/or non-steroidal anti-inflammatory drugs. One patient showed ecchymotic manifestations at the level of the anatomical snuffbox: the ecchymotic skin manifestations resolved within the following 2 weeks. At the 1-month follow-up, no patients reported hypoesthesia or dysesthesias, such as numbness and burning. No patients showed infectious complications.

## Discussion

Our experience demonstrates that an adequate number and quality of synovial biopsy samples can be safely obtained with a semi-automatic core needle introduced inside the RC joint using the anatomical snuffbox as the access route. Based on the co-primary outcomes of the study, the success rate of the procedure was 95%.

We could retrieve the predetermined number of 12 samples in 90% of the procedures and obtain gradable tissue for histopathological analyses in 19/20 biopsies, including cases displaying minimal synovial hypertrophy at local US examination. Furthermore, except for the few millimeters of the synovium lining the capsule stretched between the radius and the scaphoid, we were able to reach and perform targeted sampling in the entire synovial surface, with the constant awareness, through US guidance, of also sampling the synovium located close to the cartilage-pannus junction, both on the carpal and radial sides. This issue is relevant and may have an impact on synovial tissue research both in patients with clinically active and sub-clinical disease (remission and pre-clinical phases). Indeed, one aspect to be considered in the microscopic study of synovial biopsy specimens is intra-articular variability, as synovial inflammation may be expressed differently at different sites of the same joint. In particular, tissue samples from sites close to the cartilage-carpal junction may show different inflammatory biomarkers that may be under/overestimated when analyzing samples from more superficial joint sites [23–25]. Comparing the extent and quality of the synovial inflammatory process in different areas of the same joint may also provide spatial information on specific pathogenetic mechanisms, unraveling those of district origin, which might be more closely related to complex osteitic or enthesitic processes [26].

The entry of the biopsy instrument through the anatomical snuffbox offers ample guarantees against possible iatrogenic damage to joint tissues, especially cartilaginous and extra-articular. The needle remains sufficiently distanced from the cartilaginous floor of the carpus throughout its journey, making it difficult, in the absence of methodological errors, for the needle tip to encounter the cartilaginous surface. Ligamentous structures do not reinforce the capsular area corresponding to the anatomical snuffbox because the radial collateral ligament, which runs from the radius’s styloid process to the scaphoid’s tubercle, is covered by the abductor pollicis longus tendon. Therefore, entry through this area is not capable of damaging the ligamentous structures and should not cause joint instability, allowing a wider comfort zone for intra-articular movement.

On the other hand, there are also specific iatrogenic risks that should be considered when focusing on the area of the anatomical snuffbox: (i) the path of the radial artery and (ii) two superficial sensory branches of the radial nerve, the superficial branch and the lateral antebrachial cutaneous nerve, which are responsible for the sensitivity of the dorsum of the first finger. However, the radial artery crosses the snuffbox near the trapezius-scaphoid at an average distance of 7.5 mm from the radial styloid, and its pulsation is easily appreciable.

Similarly, the intersection of the sensory nerve trunks with the tendons delineating the anatomical snuffbox occurs distally. Therefore, the most proximal area of the anatomical snuffbox, at no more than 5 mm from the radial styloid and close to the extensor pollicis longus, represents the safest entry route, which protects both the radial artery and the sensory branches.

In 1995, Steinberg et al. [27] identified a small but substantial safe area of approximately 0.682 cm^2^ within the anatomical snuffbox near the radial styloid for percutaneous fixation of Kirschner wires. However, Korcek and Wongworawat [28] challenged this hypothesis, showing that the unpredictability of the course of the two sensory branches made it impossible to define a proper safety zone in the anatomical snuffbox. In our experience, based on the identification of the bottom of the anatomical snuffbox immediately below the extensor pollicis longus tendon and immediately proximal to the trapezius as the gateway, no neurological damage (such as hypoesthesia or dysesthesia on the dorsum of the thumb) or damage to the radial artery (in the form of significant hemorrhagic complications or aneurysmal dilatation) occurred in any of the patients. Therefore, we believe that carefully palpating the anatomical snuffbox and identifying the radial pulse (together with a preliminary US examination) is sufficient to delineate an actual safety zone for each patient and prevent iatrogenic vascular-nervous damage.

Although specific head-to-head studies are required, the safe wide range of motion allowing entry from the anatomical snuffbox is likely to offer potential advantages over the most used dorsal entry ports [13–15]. They stand in the soft tissue concavity between the tendons of the extensor pollicis longus and the extensor digitorum or between the extensor pollicis longus and the extensor carpi radialis longus or between the extensor digitorum and the extensor carpi ulnaris, approximately 1 cm distal to the Lister’s tubercle. Through these accesses, the needle enters the RC joint in a vertical direction or with an inclination conditioned by the extensor tendons, in any case not less than 45° in the radio-ulnar or ulnocarpal direction. In this way, whether a forceps or a semi-automatic core needle is used, the instrument penetrates the joint by less than 2 cm at an angle that only reaches just part of the synovial surface, missing the synovial membrane that lines the dorsal vault of the joint capsule and the extreme medial and lateral portions of the joint.

Finally, despite our proof-of-concept study allowed us to confirm a high success rate of the procedure, it is important to emphasize a limitation of the analysis that is the lack of an inter-operator variability test. Current results should be interpreted in the context of procedures performed by a single operator with > 5 years of experience in the US-guided biopsy approach.

## Conclusions

In summary, our results suggest that the use of the anatomical snuffbox as a gateway for performing a synovial biopsy of the RC joint in minimally invasive US-guided procedures allows not only to obtain adequate synovial tissue for histologic evaluation but may also offer a safe complementary tool for improved sampling and targeted analyses during single and serial procedures.

## Data Availability

Data are available upon reasonable request. Data relevant to the study are included in the article. Deidentified participant rough data are available from the corresponding author (ludovico.destefano01@universitadipavia.it) upon reasonable request.

## Abbreviations

US: Ultrasound
RC: Radiocarpal
RA: Rheumatoid arthritis
GS: Gray scale
PD: Power Doppler
H&E: Hematoxylin and eosin
IST: Intact synovial tissue
TQR: Tissue quality ratio
VAS: Visual analog scale
DAS28: 28-Joints Disease Activity Score
Δ: Delta
SD: Standard deviation
LAI: Lymphocyte aggregation index
ne: Not evaluable
